# Use of a gas-operated ventilator as a noninvasive bridging respiratory therapy in critically Ill COVID-19 patients in a middle-income country

**DOI:** 10.1007/s11739-024-03681-w

**Published:** 2024-06-28

**Authors:** Pedro P. Arias-Sanchez, Pedro D. Wendel-Garcia, Hugo A. Tirapé-Castro, Johanna Cobos, Selena X. Jaramillo-Aguilar, Arianna M. Peñaloza-Tinoco, Damary S. Jaramillo-Aguilar, Alberto Martinez, Juan Pablo Holguín-Carvajal, Enrique Cabrera, Ferran Roche-Campo, Hernan Aguirre-Bermeo

**Affiliations:** 1 Division of Pulmonary and Critical Care Medicine, Department of Medicine, Mayo Clinic, Rochester, MN USA; 2 Institute of Intensive Care Medicine, University Hospital of Zurich, Zurich, Switzerland; 3 Intensive Care Unit, Hospital Vicente Corral Moscoso, Cuenca, Ecuador; 4 Faculty of Medicine, Universidad de Cuenca, Cuenca, Ecuador; 5 Emergency Department, Hospital Vicente Corral Moscoso, Cuenca, Ecuador; 6 Intensive Care Unit, Hospital Verge de la Cinta de Tortosa, Tarragona, Spain; 7 The Pere Virgili Institute for Health Research (IISPV), Tarragona, Spain

**Keywords:** Respiratory insufficiency, COVID-19, Respiratory therapy, Noninvasive ventilation, Critical illness, Gas-operated Ventilator

## Abstract

During the COVID-19 pandemic, there was a notable undersupply of respiratory support devices, especially in low- and middle-income countries. As a result, many hospitals turned to alternative respiratory therapies, including the use of gas-operated ventilators (GOV). The aim of this study was to describe the use of GOV as a noninvasive bridging respiratory therapy in critically ill COVID-19 patients and to compare clinical outcomes achieved with this device to conventional respiratory therapies. Retrospective cohort analysis of critically ill COVID-19 patients during the first local wave of the pandemic. The final analysis included 204 patients grouped according to the type of respiratory therapy received in the first 24 h, as follows: conventional oxygen therapy (COT), n = 28 (14%); GOV, n = 72 (35%); noninvasive ventilation (NIV), n = 49 (24%); invasive mechanical ventilation (IMV), n = 55 (27%). In 72, GOV served as noninvasive bridging respiratory therapy in 42 (58%) of these patients. In the other 30 patients (42%), 20 (28%) presented clinical improvement and were discharged; 10 (14%) died. In the COT and GOV groups, 68% and 39%, respectively, progressed to intubation (P ≤ 0.001). Clinical outcomes in the GOV and NIV groups were similar (no statistically significant differences). GOV was successfully used as a noninvasive bridging respiratory therapy in more than half of patients. Clinical outcomes in the GOV group were comparable to those of the NIV group. These findings support the use of GOV as an emergency, noninvasive bridging respiratory therapy in medical crises when alternative approaches to the standard of care may be justifiable.

**Supplementary Information:**

The online version contains supplementary material available at 10.1007/s11739-024-03681-w.

## Background

During the COVID-19 pandemic, a critical gap emerged between the supply and demand for human and material resources needed to properly treat patients [1, 2]. Although the supply of equipment and resources was insufficient in many countries, including high-income countries [3, 4], this was especially evident in low- and middle-income countries (LMIC) [5, 6]. The undersupply of respiratory support devices, such as mechanical ventilators, was particularly notable [7].

**Fig. 1:**
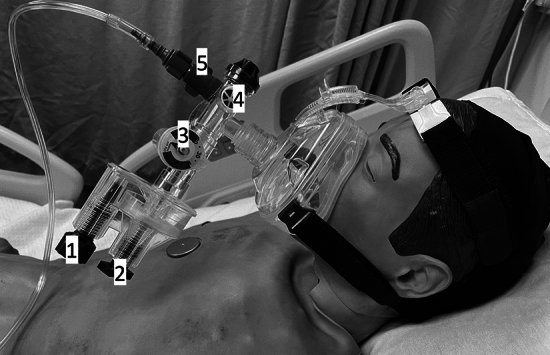
The gas operated ventilator (GO2VENT™) with an oro-nasal mask connected to an oxygen gas supply. Device components: (1) Rate dial, (2) Peak inspiratory pressure dial, (3) manometer, (4) One-way valve for entraining additional air, and (5) FiO2 control knob

Conventional respiratory therapies for critically ill COVID-19 patients include conventional oxygen therapy (COT), high-flow nasal cannula (HFNC), noninvasive ventilation (NIV), and invasive mechanical ventilation (IMV) [8]. Multiple studies have evaluated the role of these therapies in patients with COVID-19, with mixed results [9–18]. Most of these therapies require specific devices such as a ventilator and air-oxygen blender, the supply of which was limited during the pandemic, especially in LMICs. As a result, many hospitals turned to alternative respiratory therapies [7, 19–26], including gas-operated ventilators (GOV) [20] (Fig. 1), a device originally developed for the transport of intubated patients in emergency situations [27–30].

GOV is an inexpensive device that was widely available during the pandemic, even in LMICs. However, laboratory tests have found this device to be unreliable and unpredictable [31, 32] and it does not meet ARDSnet standards for invasive ventilation [33]. Despite these drawbacks, some authors suggested that GOV could be used, on an emergency basis, as a noninvasive bridging therapy in COVID-19 patients [20, 25]. However, the true value of GOV as a noninvasive bridging respiratory therapy in critically ill COVID-19 patients is not clear due to the lack of published clinical data.

During the first wave of the COVID-19 pandemic in Ecuador, the demand for ventilators and high-flow oxygen supply (> 15L/min) exceeded the supply of these devices, leading some hospitals to search for alternatives such as GOV as a noninvasive bridging therapy until HFNC, NIV and IMV became available. In this context, the aim of the present study was to describe the use of GOV as noninvasive bridging therapy in critically ill COVID-19 patients and to compare clinical outcomes achieved with GOV to conventional respiratory therapies.

## Methods

Retrospective, observational cohort study conducted at the “Vicente Corral Moscoso” hospital in Cuenca, Ecuador, a middle-income country. All data were collected during the first local wave of the COVID-19 pandemic, from July to September 2020 (**eFigure 1**).

### Study population

All patients with a positive SARS-Cov2 test (RT-PCR) admitted to the hospital were included. Patients with a COT < 10 L/min (or missing data) were excluded.

### Variables

The following demographic and clinical variables were registered: sex; age; time from symptom onset to hospitalization; body mass index (BMI); SAPS 3 score; and comorbidities.

We classified patients into four groups according to the type of respiratory therapy received during the first 24 h of admission (COT, GOV, NIV, and IMV). We recorded the first parameters measured after starting respiratory support, as follows: mean blood pressure; heart rate; respiratory rate; partial pressure of arterial oxygen (PaO2); partial arterial pressure of carbon dioxide (PaCO2); and PaO2/FiO2 (fraction of inspired oxygen).

Since GOV was used as an emergency bridging therapy, baseline clinical and laboratory parameters were available and registered prior to its use. Progression to intubation, duration of respiratory therapy, length of intensive care unit (ICU) stay, and ICU survival rates were registered.

### Hospital infrastructure during the COVID-19 pandemic

Ecuador has limited health infrastructure resources. At the onset of the COVID-19 pandemic, the reported infrastructure was 2.7 ICU beds, 3.8 ventilators, and 2.85 ICU specialists per 100,000 inhabitants [34].

At the “Vicente Corral Moscoso” hospital, all patients with a confirmed positive RT-PCR test for SARS-CoV2 were placed in an area (“the COVID-19 area”) specifically adapted for the treatment of COVID-19 patients during the pandemic. This area was divided into two sections based on disease severity: stable patients were placed in the “stable COVID-19 area”, while critically ill patients were assigned to the “ICU COVID-19 area”. The capacity of each area was increased as needed. During the first local wave, the maximum capacity was 70 beds (52 and 18 beds, respectively for stable and critically ill patients). The “stable COVID-19 area” only had low-flow (15L/min) oxygen supply. In the “ICU COVID-19 area”, four conventional respiratory therapies were available: COT, HFNC, NIV and IMV. Of the 18 critical care beds, 10 had compressed air and high-flow oxygen supplies while the remaining eight beds only had high-flow oxygen supply. Due to the limited availability of mechanical ventilators with air compressors, IMV and other conventional respiratory therapies were greatly limited. At the beginning of the first wave, a total of 20 ventilators were available, nine of which required compressed air and oxygen supply [35].

GOV can be connected to low-flow oxygen supply (15L/min), this allowed to place critically ill patients in the “stable COVID-19 area” due to the unavailability of critical care beds.

### Respiratory therapies

The respiratory therapy was selected at admission by the treating physician based on the patient’s clinical status, the physician’s expertise, and the respiratory therapies available at that time. The respiratory therapies included in the study were:

1. COT: respiratory support was provided through a reservoir mask with titratable oxygen flow between 10 – 15 L/min.
2. GOV: respiratory support was provided with the GO2VENT™ GOV model (Vortran Medical Technology 1 Inc., Sacramento, CA, USA), a single-use disposable device that provides hand-free ventilation when connected to a continuous oxygen source. This device is compatible with orifice-type flowmeters like those which are commonly used on medical oxygen cylinders with maximum flow of 15L/min. If it is connected to an in-hospital gas supply, the device limits the maximum flow rate to 40 L/min. The initial device settings were as follows: FiO2: 50%, peak inspiratory pressure (PIP) range: 20 to 40 cm H2O. The positive end-expiratory pressure (PEEP) value was expected to be 1/5 of PIP. Automatic cycling was inactivated. The gas flow source was connected only to an oxygen flow rate at 15L/min. This device has an air entrainment valve which allows the patient to entrain additional air to meet respiratory demand [36]. An oro-nasal mask interface was used in all patients (Fig. 1).
3. NIV: respiratory support with PEEP and pressure support was given through an oro-nasal mask interface. The settings were individualized by the attending physician.
4. IMV: the initial settings were as follows: tidal volumes of 6 mL/kg of predicted body weight (PBW), moderate level of PEEP [37] adjusted to the best respiratory compliance, with plateau pressure < 28 cm H2O; the respiratory rate and FiO2 levels were set according to the gas exchange.

### Statistical methods

Data are expressed as medians with interquartile ranges (IQR), or as numbers and percentages, as appropriate. We performed the Kolmogorov–Smirnov test to determine data distribution normality, which was non-normal. To compare the four respiratory therapy groups., we used either the Krustal-Wallis test or the chi-square test, as appropriate. A two-tailed p-value < 0.05 was considered statistically significant.

The Wilcoxon signed-rank test was used to compare differences before and after GOV administration. The SPSS statistical software program, v 25.0 (IBM-SPSS, Chicago, Illinois; USA) was used to perform the statistical analysis.

## Results

In the first year of the local COVID-19 pandemic, a total of 1400 patients were admitted to “the COVID-19 area”. Of these, 37% (n = 515) were critically ill patients. Two major waves of COVID-19 infections were registered during this period. The first wave occurred between July and September 2020 (**eFigure 1**).

**Fig. 2:**
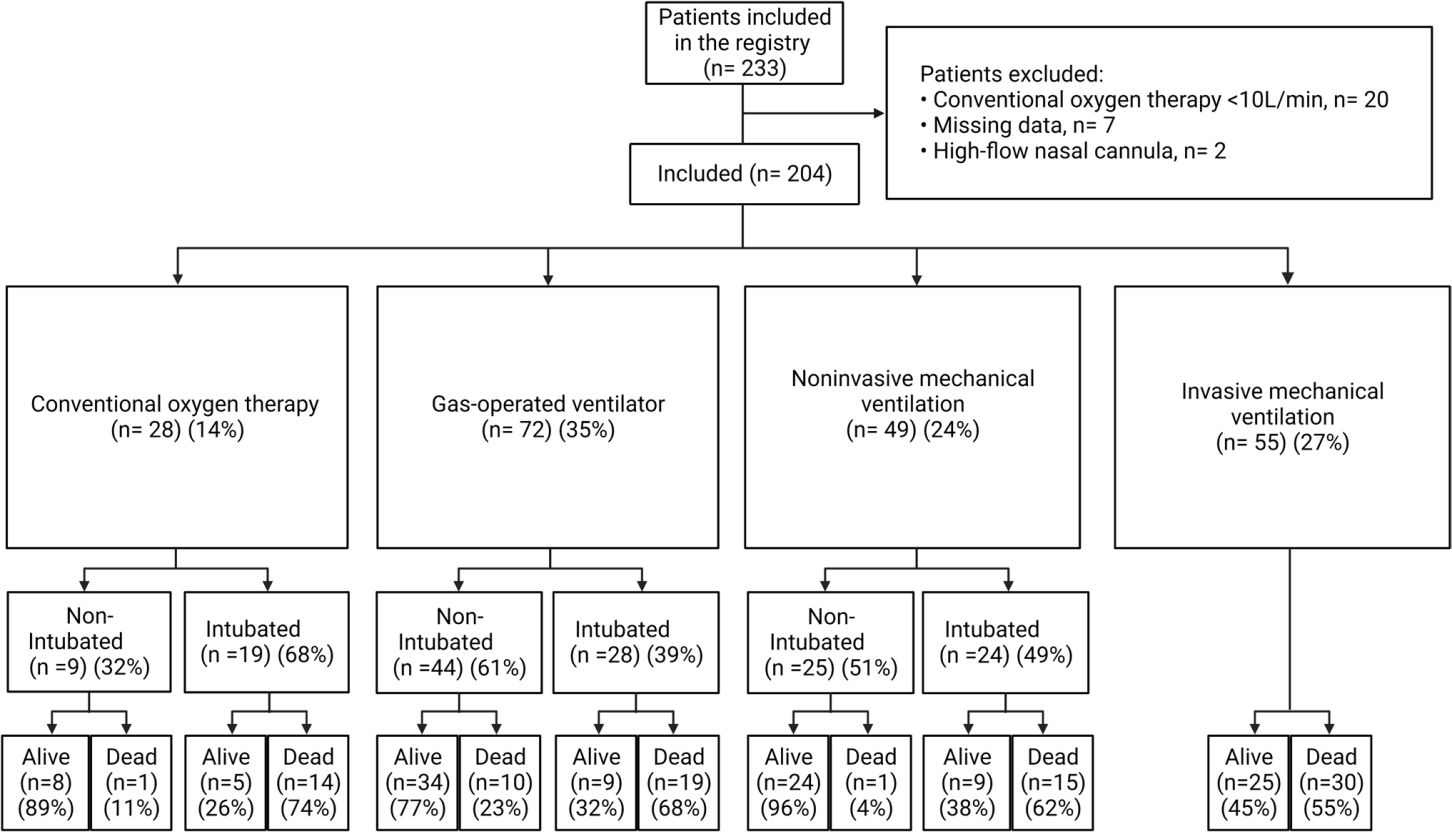
Study flowchart

During the first wave, 614 patients were admitted to “the COVID-19 area”. Of these, 233 (38%) were considered critically ill (**eFigure 1**). Of these 233 patients, twenty were excluded due to COT < 10L/min, seven due to missing data and two patients who received HFNC were also excluded due to the small sample size. Therefore, a total of 204 patients were included in the final analysis. These patients were classified into four groups according to the type of respiratory support received in the first 24 h, as follows: COT (n = 28, 14%); GOV (n = 72, 35%); NIV (n = 49, 24%); and IMV (n = 55, 27%) (Fig. 2).

Table 1 shows the clinical and demographic characteristics of the four groups at baseline. As that table shows, the only significant difference among the groups was a higher SAPS 3 score in the IMV group. Table 2 shows the hemodynamic, respiratory mechanics, and gas exchange variables of the patients according to the type of respiratory therapy received in the first 24 h of admission.

**Table 1:** - Demographic and clinical characteristics of the study sample (n = 204) at admission to the COVID-19 ICU

Variable	COT [n = 28]	GOV [n = 72]	NIV [n = 49]	IMV [n = 55]	P value	Intergroup differences
Male, n (%)	19 (68)	39 (54)	26 (53.1)	41 (75)	0.057	
Age, years	60 [42, 70]	63 [54, 74]	59 [48, 68]	62 [54, 72]	0.44	
Time from symptom onset to hospitalization, days	6 [2, 8]	7 [5. 8]	7 [5, 10]	7 [5, 10]	0.07	
BMI	26 [22, 31]	26 [24, 29]	27 [26, 28]	27 [26, 31]	0.29	
SAPS 3	59 [46, 68]	54 [42, 74]	49 [44, 64]	67 [63, 72]	< 0.001	c, e, f
Comorbidities						
Chronic Obstructive Pulmonary Disease	2 (7.1)	10 (13.9)	1 (2.1)	3 (5.6)	0.102	
Diabetes Mellitus	6 (21.4)	28 (38.9)	13 (27.1)	11 (20.4)	0.099	
Heart Failure	0 (0)	3 (4.2)	0 (0)	1 (1.9)	0.346	
Hypertension	9 (32.1)	34 (47.2)	17 (35.4)	21 (38.6)	0.435	

Data are presented as numbers (percentage) or medians [interquartile range]

Intergroup differences (p < 0.05): a, COT vs. GOV; b, COT vs. NIV; c, COT vs IMV; d, GOV vs. NIV; e, GOV vs. IMV; f, NIV vs. IMV

*COT* Conventional oxygen therapy, GOV  Gas-operated ventilator, NIV  Non-invasive mechanical ventilation, *IMV* Invasive mechanical ventilation

**Table 2:** Clinical and gas-exchange parameters in the first 24 h of COVID-19 ICU admission, progression to IMV, and clinical outcomes

Variable	COT [n = 28]	GOV [n = 72]	NIV [n = 49]	IMV [n = 55]	P value	Intergroup differences
Mean blood pressure, mmHg	93 [85, 100]	87 [77, 94]	85 [80, 95]	81 [73, 95]	0.07	
Heart rate, beats/min	92 [80, 106]	96 [88, 106]	78 [72, 82]	78 [70, 90]	< 0.001	b, c, d, e
Respiratory rate, breaths/min	32 [24, 38]	25 [24, 28]	40 [32, 42]	35 [28, 40]	< 0.001	a, b, d, e
PaO2, mmHg	54 [47, 63]	58 [53, 67]	58 [53, 65]	68 [59, 79]	< 0.001	c, e, f
PaCO2, mmHg	34 [31, 38]	35 [29, 37]	35 [32, 38]	41 [33, 50]	< 0.001	c, e, f
PaO2/FiO2, mmHg	96 [61, 119]	116 [106, 133]	117 [96, 164]	116 [96, 156]	0.01	a, b, c
Duration of Respiratory therapy, days	1 [1]	1 [1, 3]	2 [1, 3]	6 [4, 9]	< 0.001	b, c, e, f
Progression toward IMV, n (%)	19 (68)	28 (39)	24 (49)	–	0.03	a
ICU length of stay, days	10 [7, 16]	8 [5, 11]	10 [5, 13]	12 [8, 16]	< 0.001	e
ICU survival, n (%)	13 (46)	43 (60)	33 (67)	25 (45)	0.09	

Data are presented as numbers (percentage) or medians [interquartile range]

Intergroup differences (p < 0.05): a, COT vs. GOV; b, COT vs. NIV; c, COT vs IMV; d, GOV vs. NIV; e, GOV vs. IMV; f, NIV vs. IMV

*COT*  Conventional oxygen therapy, *GOV*  Gas-operated ventilator, *NIV*  Non-invasive mechanical ventilation, *IMV*  Invasive mechanical ventilation, *ICU*  Intensive Care Unit

Seventy-two patients received GOV as the initial respiratory therapy. In 42 (58%) of these patients, GOV was used as a noninvasive bridging respiratory therapy. These 42 patients were later switched to HFNC (n = 6, 8%), NIV (n = 8, 11%), or IMV (n = 28, 39%). In the remaining 30 (42%) patients, GOV was the only respiratory therapy used. Of those 30 patients, 20 (28%) showed clinical improvement and were switched to COT (< 10L/min) and discharged from “the COVID-19 area”; the remaining 10 (14%) patients died (**eFigure 2**).

There were no significant differences for any of the study variables between the COT and GOV groups prior to initiation of GOV (Table 3). Following initiation of GOV, the patients showed significant improvement on all hemodynamic, respiratory mechanics, and blood gas exchange parameters (Table 4). GOV was associated with significantly lower intubation rates than COT (39% vs. 68% p = 0.009).

**Table 3:** Comparison of clinical characteristics and gas-exchange parameters at O2 of 15L/min between the COT and GOV groups (prior to starting GOV)

Variable	COT [n = 28]	Before GOV [n = 72]	P value
Mean blood pressure, mmHg	92.5 [85, 100]	89 [82, 98]	0.322
Heart rate, beats/min	92 [80, 106]	102 [88, 116]	0.047
Respiratory rate, breaths/min	32 [24, 37.5]	28 [26, 32]	0.298
PaO2, mmHg	54 [46.5, 63]	54 [48, 63]	0.954
PaCO2, mmHg	33.7 [30.7, 37.5]	32 [28, 39]	0.342
PaO2/FiO2	95.6 [61.4, 119.3]	87 [69, 107.8]	0.477

Data are presented as medians [interquartile range]

*COT* Conventional oxygen therapy, *GOV*  Gas-operated ventilator

**Table 4:** Clinical characteristics and gas-exchange parameters in the GOV group before and after starting gas operated ventilator therapy

Variables	Before GOV [n = 72]	After GOV* [n = 72]	P value
Mean blood pressure, mmHg	89 [82, 98]	87 [77, 94]	0.002
Heart rate, beats/min	102 [88, 116]	96 [88, 106]	< 0.001
Respiratory rate, breaths/min	28 [26, 32]	25 [24, 28]	< 0.001
PaO2, mmHg	54 [48, 63]	58 [53, 67]	0.002
PaCO2, mmHg	32 [28, 39]	35 [29, 38]	0.31
PaO2/FiO2, mmHg	87 [69, 108]	116 [106, 133]	< 0.001

Data are presented as medians [interquartile range]

*GOV*  Gas-operated ventilator

^*^ The first available parameters measured after starting the respiratory support on the first day of admission

No significant differences in clinical outcomes were observed between the GOV and NIV groups (Table 2). The length of ICU stay was shorter in the GOV group compared to IMV (8 days [5–11] vs. 12 days [8–16], p = 0.001).

A substantial proportion of patients in all groups progressed to intubation, as follows: COT (n = 19, 68%); GOV (n = 28, 39%); and NIV (n = 24, 49%). No significant differences in ICU survival (p = 0.42) or ICU length of stay (p = 0.56) were observed between these groups and the IMV group. (**eTable 1**). The supplemental digital document shows the characteristics of the groups who progressed to intubation (**eTable 1, eTable 2**) and the characteristics of the patients according to survival outcome (**eTable 3, eTable 4**).

## Discussion

This retrospective study was conducted in a group of critically ill COVID-19 patients treated at a hospital located in a middle-income country. GOV was successfully used as a noninvasive bridging respiratory therapy in more than half (58%) of the patients. Intubation rates were lower in the patients who received GOV compared to those who underwent COT. Interestingly, there were no statistically significant differences in clinical outcomes between the GOV and NIV groups. Moreover, there were no significant differences in ICU survival rates between GOV and the conventional respiratory therapies. To our knowledge, this is the first clinical study to evaluate GOV as a noninvasive bridging respiratory therapy and to compare clinical outcomes to COT, NIV, and IMV.

### Gas-operated ventilator as a bridging respiratory therapy

During the COVID-19 pandemic, when mechanical ventilators and related supplies were absent or highly limited, several alternatives were proposed as bridging respiratory therapies, especially in developing countries [7, 19–22]. For example, one proposal suggested that two patients on IMV could share the same ventilator [38], although this idea was discouraged by experts [24]. In addition, new devices were developed through joint collaborations between industry and academic institutions, including the UCL Ventura, a noninvasive continuous positive airway pressure (CPAP) device made by UCL and Mercedes-AMG HPP [22]. In Ecuador, the only available alternative device was the GOV. This device had been previously tested in laboratory settings for use in IMV; however, given the highly variable performance of this device, it was only recommended for use under continuous monitoring by a trained operator [27]. Nonetheless, it is important to underscore that gas-operated ventilators have been successfully used in humans during hospital transport and in certain emergency situations, such as loss of backup power to a hospital during Hurricane Katrina [20, 27, 28, 31].

Carcamo et al. conducted a feasibility study to evaluate the UCL Ventura in a sample of 45 COVID-19 patients who had previously required high-concentration oxygen (15L/min). They used the UCL-Ventura “Wayrachi CPAP”, which was reverse-engineered from the Phillips Respironics™ whisperflow device [39]. Although the UCL-Ventura device was successfully used as a noninvasive bridging respiratory therapy to IMV in 30% of patients. By contrast, in our cohort, GOV had a substantially higher success rate (58%). Moreover, more than one in four patients (28%) in our sample improved sufficiently to be discharged from the “ICU COVID-19 area" using GOV as the only noninvasive respiratory therapy.

As mentioned earlier, these devices have been useful in disaster medicine. Consequently, we suggest that our data can be used to provide constructive guidance to clinicians in the arena of disaster medicine, such as the COVID-19 pandemic, when deviation from the standard of care can be deemed justifiable.

### Gas-operated ventilator vs. conventional oxygen therapy

Numerous studies have compared respiratory therapies (HFNC and/or NIV) to COT in terms of intubation and mortality rates, with mixed results [9, 10, 12, 40, 41]. GOV has not been previously compared to COT.

Although the baseline characteristics (i.e., prior to starting GOV) of the patients who received GOV and COT were similar (Table 3), the use of GOV improved all clinical characteristics and gas-exchange parameters (Table 4). This beneficial effect could potentially improve clinical outcomes, as suggested by the intubation rates in the GOV and COT group, in which only 39% of those who received GOV progressed to IMV versus 68% of those who received COT, a statistically significant difference. In addition, ICU survival rates were higher in the GOV group versus COT (60% vs. 46%, respectively), although this difference was not statistically significant. The lower intubation rate observed in the GOV group may be due to the improvement in gas-exchange parameters, although more data are needed to confirm this.

### Gas-operated ventilator vs. noninvasive ventilation

Several studies involving COVID-19 patients with acute respiratory failure have compared NIV to other noninvasive respiratory therapies, with heterogenous findings [10, 11, 13, 14, 18]. However, clinical studies comparing GOV to NIV are lacking. When the availability of conventional respiratory therapies is limited, GOV is an attractive alternative to more advanced techniques. Theoretically, the respiratory support provided by GOV should be similar to that achieved with NIV because the device provides a constant, cyclical flow pressure that generates both PIP and PEEP [36].

When we compared GOV to NIV, we did not find statistically significant differences in respiratory mechanics or gas exchange parameters between the two respiratory therapies. These data support the use of GOV as a feasible noninvasive respiratory therapy because the clinical outcomes are comparable to NIV, with the additional advantages of being easy to use and compatible with widely used medical oxygen cylinders that can deliver 15L/min.

### Gas-operated ventilator vs. invasive mechanical ventilation

In the present study, we found that these two techniques differed significantly in terms of respiratory mechanics and gas exchange parameters; however, these findings should be interpreted cautiously given the small sample size. The protective ventilation and the effect of a higher PEEP provided by IMV could explain these findings. Importantly, patients on GOV spent significantly fewer days in the ICU compared to the IMV group (median 8 vs. 12 days, respectively). Despite these differences, we found no significant differences in ICU survival rates between these two groups.

An observational study performed by Siempos et al. [42] found that early (≤ 24 h of ICU admission) vs. delayed intubation had no significant impact on ICU survival or length of stay. This finding was subsequently confirmed in the meta-analysis (12 studies) by Papoutsi et al. [15]. However, other studies have found that delayed intubation is associated with an increase in morbidity and mortality [16, 17, 43–45]. In our hospital, due to the mismatch between the supply of ventilators and the demand for IMV, early intubation was, in many cases, not feasible, which is why GOV was considered as an alternative. Although Reyes et al. [46] previously reported that patients who failed NIV or HFNC and progressed to IMV had lower survival rates, we did not find any significant differences in ICU survival rates between patients who failed GOV and had to be intubated vs. those who received IMV as the initial treatment (32% vs. 45%, p = 0.164). In fact, among patients who progressed to IMV, we did not find statistical differences between GOV and other conventional respiratory therapies in terms of ICU survival, duration of respiratory therapy, and ICU length of stay (eTable1). Moreover, among patients who did not progress to IMV, the GOV group had a shorter duration of respiratory therapy than the NIV group (2 [1, 3] vs. 3 [2, 4], p = 0.012) (eTable2).

When we analyzed the patients who died, we did not find statistical differences in the main outcomes between the GOV group and conventional respiratory therapies (eTable3). Additionally, patients in the GOV group had lower, but not statistically significant, progression rates toward IMV compared with the COT and NIV groups (66% vs. 93% and 94%, respectively) (eTable3). This trend may be explained by limited access to IMV during this medical crisis.

## Limitations

This study has limitations associated with the retrospective study design and the use of patient registry data obtained during a period (COVID-19 pandemic) when human and material resources were limited. First, we were unable to determine the number of patients in the GOV group that did not have access to a mechanical ventilator. Second, we do not have data on clinical tolerance to GOV. Third, given the retrospective nature of the study, additional data on complications such as pulmonary thromboembolism, multiorgan failure, acute kidney injury, and cardiac injury are not available. Fourth, the small sample size and the specific characteristics of these patients (representing the initial wave before vaccines were accessible), necessitates a cautious approach to interpreting the comparisons. By contrast, an important strength is that this is the first clinical study to evaluate GOV as a noninvasive bridging respiratory and to compare clinical outcomes achieved with this device to conventional respiratory therapies. This provides a valuable foundation to support further research to confirm these results in clinical populations with similar characteristics.

## Conclusion

In this sample of critically ill COVID-19 patients in a middle-income country with limited resources, GOV was successfully used as a noninvasive bridging respiratory therapy in more than half of the patients. Moreover, there were no significant differences in clinical outcomes between patients who received GOV and those who underwent NIV. These promising results support GOV as an emergency, noninvasive bridging respiratory therapy in medical crises when alternative approaches to standard of care may be justifiable.

## Supplementary Information

Below is the link to the electronic supplementary material.

Supplementary file1 (DOCX 100 KB)

## Data Availability

The datasets used and/or analyzed during the current study are available from the corresponding author on reasonable request.
