# Correction: Use of a gas-operated ventilator as a noninvasive bridging respiratory therapy in critically Ill COVID-19 patients in a middle-income country

**DOI:** 10.1007/s11739-024-03703-7

**Published:** 2024-07-13

**Authors:** Pedro P. Arias-Sanchez, Pedro D. Wendel-Garcia, Hugo A. Tirapé-Castro, Johanna Cobos, Selena X. Jaramillo-Aguilar, Arianna M. Peñaloza-Tinoco, Damary S. Jaramillo-Aguilar, Alberto Martinez, Juan Pablo Holguín-Carvajal, Enrique Cabrera, Ferran Roche-Campo, Hernan Aguirre-Bermeo

**Affiliations:** 1 Division of Pulmonary and Critical Care Medicine, Department of Medicine, Mayo Clinic, Rochester, MN USA; 2 Institute of Intensive Care Medicine, University Hospital of Zurich, Zurich, Switzerland; 3 Intensive Care Unit, Hospital Vicente Corral Moscoso, Cuenca, Ecuador; 4 Faculty of Medicine, Universidad de Cuenca, Cuenca, Ecuador; 5 Emergency Department, Hospital Vicente Corral Moscoso, Cuenca, Ecuador; 6 Intensive Care Unit, Hospital Verge de la Cinta de Tortosa, Tarragona, Spain; 7 The Pere Virgili Institute for Health Research (IISPV), Tarragona, Spain

**Correction: Internal and Emergency Medicine** 10.1007/s11739-024-03681-w

In this article the affiliation details for Author Hernan Aguirre-Bermeo were incorrectly given one affiliation as

‘Intensive Care Unit, Hospital Vicente Corral Moscoso, Cuenca, Ecuador’

but they has two affiliations should have been

‘Intensive Care Unit, Hospital Vicente Corral Moscoso, Cuenca, Ecuador’ and

‘Faculty of Medicine, Universidad de Cuenca, Cuenca, Ecuador’

The original article has been corrected.

