# Supplementary material for: Use of a gas-operated ventilator as a noninvasive bridging respiratory therapy in critically Ill COVID-19 patients in a middle-income country
Source: Intern Emerg Med. 2024 Jun 28;20(2):543–51. doi: 10.1007/s11739-024-03681-w (PMC11950081; doi:10.1007/s11739-024-03681-w)
Supplement: Supplementary file 1 — Supplementary file1 (DOCX 100 KB) [file 11739_2024_3681_MOESM1_ESM.docx]

**On-line Supplementary Appendix**

Use of a Gas-Operated Ventilator as a Noninvasive Bridging Respiratory Therapy in Critically Ill COVID-19 Patients in a Middle-Income Country

**List of Contents**

Page 3: **eTable 1**: Clinical and gas-exchange parameters in the first 24 hours of COVID-19 ICU admission in patients who progressed to IMV.

Page 3: **eTable 2**: Clinical and gas-exchange parameters in the first 24 hours of COVID-19 ICU admission in patients who did not progress to IMV.

Page 4: **eTable 3**: Clinical and gas-exchange parameters in the first 24 hours of COVID-19 ICU admission in patients who died.

Page 4: **eTable 4**: Clinical and gas-exchange parameters in the first 24 hours of COVID-19 ICU admission in patients who survived.
Page 5: **eFigure 1**: Number of hospital and ICU admissions for COVID-19 in the first year.

Page 5: **eFigure 2**: Progression in patients who received respiratory therapy with a gas-operated ventilator.

Page 6-8: **STROBE Checklist**

**Tables**

| **eTable 1. - Clinical and gas-exchange parameters in the first 24 hours of COVID-19 ICU admission in patients who progressed to IMV.** | | | | | |
| --- | --- | --- | --- | --- | --- |
| **Variable** | **COT**  **[n=19]** | **GOV**  **[n=28]** | **NIV**  **[n=24]** | **P value** | **Intergroup**  **differences** |
| Mean blood pressure, mmHg | 93 [85, 100] | 87 [78, 95] | 84 [80, 111] | 0.222 |  |
| Heart rate, beats/min | 93 [81, 116] | 104 [86, 112] | 78 [72, 84] | <0.001 | b, c |
| Respiratory rate, breaths/min | 32 [25, 38] | 27 [24, 28] | 40 [30, 42] | <0.001 | b, c |
| PaO2, mmHg | 54 [46, 59] | 56 [47, 64] | 55 [52, 59] | 0.586 |  |
| PaCO2, mmHg | 32 [23, 35] | 34 [29, 38] | 35 [31, 38] | 0.184 |  |
| PaO2/FiO2, mmHg | 73 [53, 113] | 112 [93, 128] | 102 [86, 137] | 0.003 | a, b |
| Duration of Respiratory therapy, days | 1 [1, 1] | 1 [1,2] | 1 [1, 2] | 0.058 |  |
| ICU length of stay, days | 13 [8, 19] | 11 [8, 15] | 13 [10, 21] | 0.366 |  |
| ICU survival, n (%) | 5 (26) | 9 (32) | 9 (38) | 0.738 |  |
| **Data are presented as numbers (percentage) or medians [interquartile range].**  **Intergroup differences (p < 0.05): a, COT vs. GOV; b, COT vs. NIV; c, GOV vs. NIV.**  **Abbreviations: COT = Conventional oxygen therapy; GOV = Gas-operated ventilator; NIV = Non-invasive mechanical ventilation, ICU = Intensive Care Unit** | | | | | |

| **eTable 2. - Clinical and gas-exchange parameters in the first 24 hours of COVID-19 ICU admission in patients who did not progress to IMV.** | | | | | |
| --- | --- | --- | --- | --- | --- |
| **Variable** | **COT**  **[n=9]** | **GOV**  **[n=44]** | **NIV**  **[n=25]** | **P value** | **Intergroup**  **differences** |
| Mean blood pressure, mmHg | 90 [81, 98] | 87 [76, 94] | 85 [81, 85] | 0.659 |  |
| Heart rate, beats/min | 85 [73, 99] | 94 [89, 104] | 78 [72, 79] | <0.001 | b, c |
| Respiratory rate, breaths/min | 32 [24, 37] | 24 [22, 26] | 42 [40, 42] | <0.001 | c |
| PaO2, mmHg | 62 [47, 69] | 60 [54, 67] | 59 [55, 68] | 0.975 |  |
| PaCO2, mmHg | 37 [34, 51] | 35 [29, 37] | 34 [32, 37] | 0.122 |  |
| PaO2/FiO2, mmHg | 123 [102, 132] | 120 [109, 134] | 129 [107, 168] | 0.268 |  |
| Duration of Respiratory therapy, days | 1 [1, 2] | 2 [1,3] | 3 [2, 4] | <0.001 | b, c |
| ICU length of stay, days | 6 [6, 9] | 7 [4, 9] | 6 [4, 10] | 0.862 |  |
| ICU survival, n (%) | 8 (89) | 34 (77) | 24 (96) | 0.109 |  |
| **Data are presented as numbers (percentage) or medians [interquartile range].**  **Intergroup differences (p < 0.05): a, COT vs. GOV; b, COT vs. NIV; c, GOV vs. NIV.**  **Abbreviations: COT = Conventional oxygen therapy; GOV = Gas-operated ventilator; NIV = Non-invasive mechanical ventilation, ICU = Intensive Care Unit** | | | | | |

| **eTable 3. - Clinical and gas-exchange parameters in the first 24 hours of COVID-19 ICU admission in patients who died.** | | | | | | |
| --- | --- | --- | --- | --- | --- | --- |
| **Variable** | **COT**  **[n=15]** | **GOV**  **[n=29]** | **NIV**  **[n=16]** | **IMV**  **[n=30]** | **P value** | **Intergroup**  **differences** |
| Mean blood pressure, mmHg | 93 [84, 99] | 84 [69, 94] | 90 [82, 117] | 80 [74, 95] | 0.078 |  |
| Heart rate, beats/min | 85 [80, 107] | 104 [86, 113] | 78 [72, 81] | 78 [72, 92] | <0.001 | d, e |
| Respiratory rate, breaths/min | 32 [24, 38] | 26 [24, 30] | 38 [30, 42] | 35 [30, 40] | <0.001 | d, e |
| PaO2, mmHg | 53 [44, 60] | 56 [47, 67] | 55 [52, 61] | 70 [62, 79] | <0.001 | c, e, f |
| PaCO2, mmHg | 33 [24, 39] | 35 [30, 36] | 36 [32, 42] | 39 [32, 49] | 0.031 | e |
| PaO2/FiO2, mmHg | 69 [51, 113] | 112 [93, 134] | 95 [76, 111] | 114 [94, 155] | 0.010 | a, c |
| Duration of Respiratory therapy, days | 1 [1, 1] | 1 [1,2] | 1 [1, 2] | 8 [5, 10] | <0.001 | c, e, f |
| Progression toward IMV, n (%) | 14 (93) | 19 (66) | 15 (94) | 30 (100) | 0.001 | e |
| ICU length of stay, days | 13 [8, 19] | 10 [5, 14] | 14 [9, 21] | 13 [8, 17] | 0.100 |  |
| **Data are presented as numbers (percentage) or medians [interquartile range].**  **Intergroup differences (p < 0.05): a, COT vs. GOV; b, COT vs. NIV; c, COT vs IMV; d, GOV vs. NIV; e, GOV vs. IMV; f, NIV vs. IMV.**  **Abbreviations: COT = Conventional oxygen therapy; GOV = Gas-operated ventilator; NIV = Non-invasive mechanical ventilation; IMV = Invasive mechanical ventilation, ICU = Intensive Care Unit** | | | | | | |

| **eTable 4. - Clinical and gas-exchange parameters in the first 24 hours of COVID-19 ICU admission in patients who survived.** | | | | | | |
| --- | --- | --- | --- | --- | --- | --- |
| **Variable** | **COT**  **[n=13]** | **GOV**  **[n=43]** | **NIV**  **[n=33]** | **IMV**  **[n=25]** | **P value** | **Intergroup**  **differences** |
| Mean blood pressure, mmHg | 92 [85, 100] | 89 [83, 94] | 81 [80, 86] | 81 [70, 96] | 0.161 |  |
| Heart rate, beats/min | 98 [85, 111] | 93 [89, 102] | 80 [70, 91] | 80 [70, 91] | <0.001 | b, c, d, e |
| Respiratory rate, breaths/min | 32 [25, 37] | 24 [22, 26] | 42 [36, 42] | 35 [26, 41] | <0.001 | d, e |
| PaO2, mmHg | 59 [48, 67] | 60 [56, 67] | 59 [54, 66] | 63 [52, 79] | 0.580 |  |
| PaCO2, mmHg | 35 [32, 39] | 35 [29, 38] | 33 [32, 37] | 42 [34, 53] | 0.002 | e, f |
| PaO2/FiO2, mmHg | 111 [87, 125] | 119 [112, 133] | 129 [106, 168] | 124 [97, 162] | 0.102 |  |
| Duration of Respiratory therapy, days | 1 [1, 1] | 1 [2, 3] | 3 [2, 3] | 5 [3, 8] | <0.001 | b, c, e, f |
| Progression toward IMV, n (%) | 5 (39) | 9 (21) | 9 (27) | 25 (100) | <0.001 | c, e, f |
| ICU length of stay, days | 8 [6, 11] | 8 [5, 10] | 7 [5, 11] | 10 [9, 16] | 0.005 |  |
| **Data are presented as numbers (percentage) or medians [interquartile range].**  **Intergroup differences (p < 0.05): a, COT vs. GOV; b, COT vs. NIV; c, COT vs IMV; d, GOV vs. NIV; e, GOV vs. IMV; f, NIV vs. IMV.**  **Abbreviations: COT = Conventional oxygen therapy; GOV = Gas-operated ventilator; NIV = Non-invasive mechanical ventilation; IMV = Invasive mechanical ventilation, ICU = Intensive Care Unit** | | | | | | |

**Figures**

**
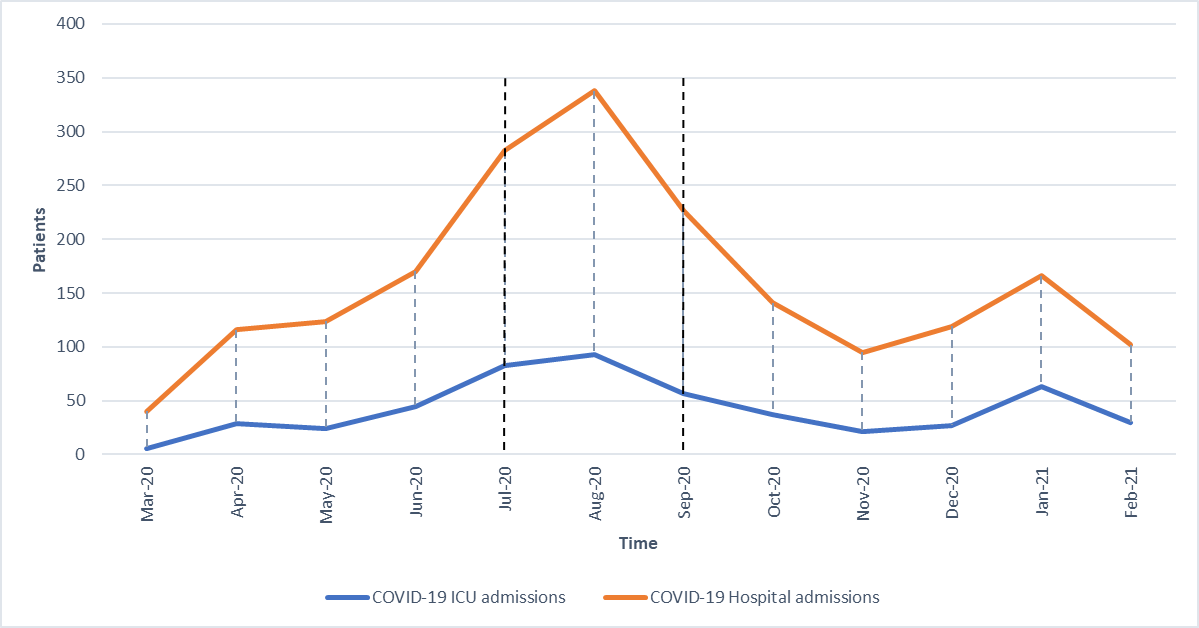
**

**eFigure 1. Number of hospital and ICU admissions for COVID-19 in the first year.**

**
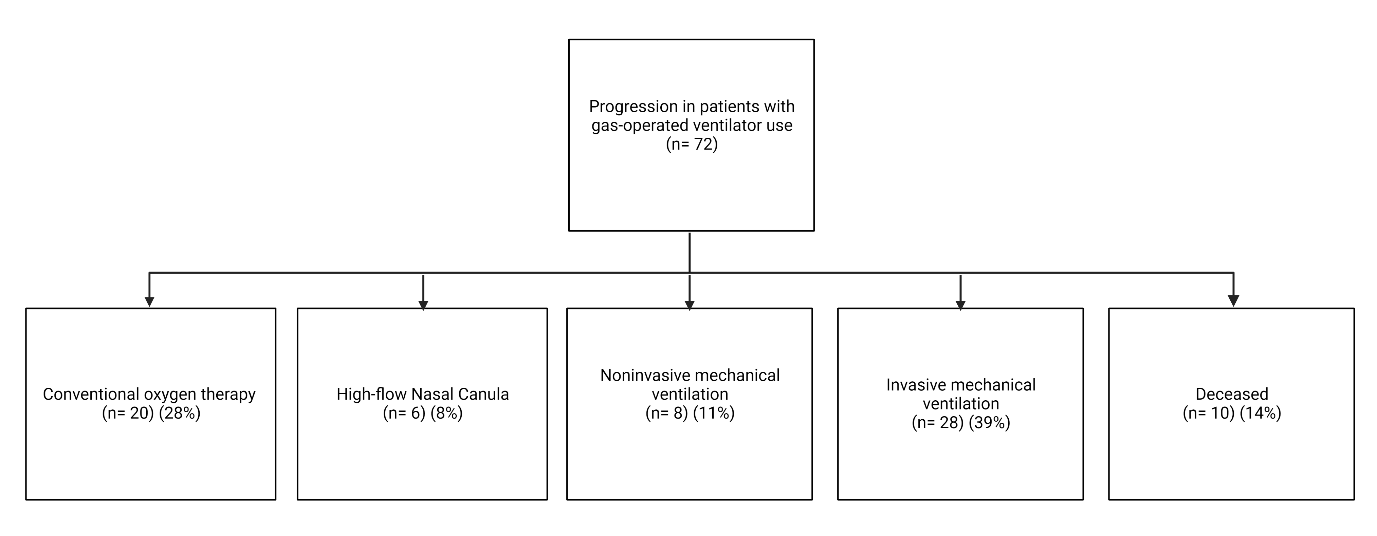
**

**eFigure 2. Progression in patients who received respiratory therapy with a gas-operated ventilator.^*^**

**^*^**Images created with BioRender.

**STROBE Statement—Checklist of items that should be included in reports of cohort studies**

|  | Item No | Recommendation | Page |
| --- | --- | --- | --- |
| **Title and abstract** | 1 | (*a*) Indicate the study’s design with a commonly used term in the title or the abstract | Title  Page 1 |
|  |  | (*b*) Provide in the abstract an informative and balanced summary of what was done and what was found | Abstract  Page 3-4 |
| Introduction | | |  |
| Background/rationale | 2 | Explain the scientific background and rationale for the investigation being reported | Background  Page 5-6 |
| Objectives | 3 | State specific objectives, including any prespecified hypotheses | Background  Page 5-6 |
| Methods | | |  |
| Study design | 4 | Present key elements of study design early in the paper | Methods  Page 6-9 |
| Setting | 5 | Describe the setting, locations, and relevant dates, including periods of recruitment, exposure, follow-up, and data collection | Methods  Page 6-9 |
| Participants | 6 | (*a*) Give the eligibility criteria, and the sources and methods of selection of participants. Describe methods of follow-up | Methods  Page 6-9 |
|  |  | (*b*) For matched studies, give matching criteria and number of exposed and unexposed | NA |
| Variables | 7 | Clearly define all outcomes, exposures, predictors, potential confounders, and effect modifiers. Give diagnostic criteria, if applicable | Methods  Page 6-9 |
| Data sources/ measurement | 8* | For each variable of interest, give sources of data and details of methods of assessment (measurement). Describe comparability of assessment methods if there is more than one group | Methods  Page 6-9 |
| Bias | 9 | Describe any efforts to address potential sources of bias | Discussion  Page 11-15 |
| Study size | 10 | Explain how the study size was arrived at | Results  Page 9-11  Figure 3 |
| Quantitative variables | 11 | Explain how quantitative variables were handled in the analyses. If applicable, describe which groupings were chosen and why | Methods  Page 6-9 |
| Statistical methods | 12 | (*a*) Describe all statistical methods, including those used to control for confounding | Methods  Page 6-9 |
|  |  | (*b*) Describe any methods used to examine subgroups and interactions | Methods  Page 6-9 |
|  |  | (*c*) Explain how missing data were addressed | Methods  Page 6-9 |
|  |  | (*d*) If applicable, explain how loss to follow-up was addressed | Methods  Page 6-9 |
|  |  | (*e*) Describe any sensitivity analyses |  |
| Results | | |  |
| Participants | 13* | (a) Report numbers of individuals at each stage of study—eg numbers potentially eligible, examined for eligibility, confirmed eligible, included in the study, completing follow-up, and analysed | Results  Page 9-11  Figure 2 |
|  |  | (b) Give reasons for non-participation at each stage | Results  Page 9-11  Figure 2 |
|  |  | (c) Consider use of a flow diagram | Figure 2 |
| Descriptive data | 14* | (a) Give characteristics of study participants (eg demographic, clinical, social) and information on exposures and potential confounders | Results  Page 9-11  Table 1 |
|  |  | (b) Indicate number of participants with missing data for each variable of interest | Results  Page 9-11 |
|  |  | (c) Summarise follow-up time (eg, average and total amount) | Results  Table 2 |
| Outcome data | 15* | Report numbers of outcome events or summary measures over time | Results  Page 9-11  Table 2, 3, 4, eTable 1 |
| Main results | 16 | (*a*) Give unadjusted estimates and, if applicable, confounder-adjusted estimates and their precision (eg, 95% confidence interval). Make clear which confounders were adjusted for and why they were included | Results  Page 9-11  Table 1, 2, 3, 4, eTable 1, eTable 2, eTable 3, eTable 4 |
|  |  | (*b*) Report category boundaries when continuous variables were categorized | NA |
|  |  | (*c*) If relevant, consider translating estimates of relative risk into absolute risk for a meaningful time period | NA |
| Other analyses | 17 | Report other analyses done—eg analyses of subgroups and interactions, and sensitivity analyses | Results  Page 9-11  Table 3, 4. |
| Discussion | | |  |
| Key results | 18 | Summarise key results with reference to study objectives | Discussion  Pages 11-16 |
| Limitations | 19 | Discuss limitations of the study, taking into account sources of potential bias or imprecision. Discuss both direction and magnitude of any potential bias | Discussion  Pages 11-16 |
| Interpretation | 20 | Give a cautious overall interpretation of results considering objectives, limitations, multiplicity of analyses, results from similar studies, and other relevant evidence | Discussion  Pages 11-16 |
| Generalisability | 21 | Discuss the generalisability (external validity) of the study results | Discussion  Pages 11-16 |
| Other information | | |  |
| Funding | 22 | Give the source of funding and the role of the funders for the present study and, if applicable, for the original study on which the present article is based | Page 17 |

*Give information separately for exposed and unexposed groups.

**Note:** An Explanation and Elaboration article discusses each checklist item and gives methodological background and published examples of transparent reporting. The STROBE checklist is best used in conjunction with this article (freely available on the Web sites of PLoS Medicine at http://www.plosmedicine.org/, Annals of Internal Medicine at http://www.annals.org/, and Epidemiology at http://www.epidem.com/). Information on the STROBE Initiative is available at http://www.strobe-statement.org.
